# Integrative eQTL and Mendelian randomization analysis reveals key genetic markers in mesothelioma

**DOI:** 10.1186/s12931-025-03219-4

**Published:** 2025-04-13

**Authors:** Jinsong Li, Xingmeng Wang, Yaru Lin, Zhengliang Li, Wei Xiong

**Affiliations:** 1 Department of Biochemistry and Molecular Biology, College of Basic Medical Sciences, Dali University, Dali, Yunnan China; 2 Key Laboratory of Clinical Biochemistry Testing in Universities of Yunnan Province, College of Basic Medical Sciences, Dali University, Dali, Yunnan China; 3 Yunnan Provincial Key Laboratory of Entomological Biopharmaceutical R&D, College of Pharmacy, Dali University, Dali, Yunnan China; 4 Department of Radiology, The First Affiliated Hospital of Dali University, Dali University, Dali, Yunnan China

**Keywords:** eQTL localization, Mesothelioma, Mendelian randomization, Biomarkers, Tumor immunity

## Abstract

**Background:**

Mesothelioma is a rare cancer that originates from the pleura and peritoneum, with its incidence increasing due to asbestos exposure. Patients are frequently diagnosed at advanced stages, resulting in poor survival rates. Therefore, the identification of molecular markers for early detection and diagnosis is essential.

**Methods:**

Three mesothelioma datasets were downloaded from the GEO database for differential gene expression analysis. Instrumental variables (IVs) were identified based on expression quantitative trait locus (eQTL) data for Mendelian randomization (MR) analysis using mesothelioma Genome-Wide Association Study (GWAS) data from the FINNGEN database. The intersecting genes from MR-identified risk genes and differentially expressed genes were identified as key co-expressed genes for mesothelioma. Functional enrichment analyses, including Gene Ontology (GO), Kyoto Encyclopedia of Genes and Genomes (KEGG), and Gene Set Enrichment Analysis (GSEA), as well as immune cell correlation analysis, were performed to elucidate the roles of key genes in mesothelioma. Additionally, the differential expression of key genes in mesothelioma was validated in independent GEO datasets and TCGA datasets. This integrative research combining multiple databases and analytical methods established a robust model for identifying mesothelioma risk genes.

**Results:**

The research conducted in our study identified 1608 genes that were expressed differentially in mesothelioma GEO datasets. By combining these genes with 192 genes from MR analysis, we identified 14 key genes. Notably, MPZL1, SOAT1, TACC3, and CYBRD1 are linked to a high risk of mesothelioma, while TGFBR3, NDRG2, EPAS1, CPA3, MNDA, PRKCD, MTUS1, ALOX15, LRRN3, and ITGAM are associated with a lower risk. These genes were found to be enriched in pathways associated with superoxide metabolism, cell cycle regulation, and proteasome function, all of which are linked to the development of mesothelioma. Noteworthy observations included a significant infiltration of M1 macrophages and CD4 + T cells in mesothelioma, with genes SOAT1, MNDA, and ITGAM showing a positive correlation with the level of M1 macrophage infiltration. Furthermore, the differential expression analyses conducted on the GEO validation set and TCGA data confirmed the significance of the identified key genes.

**Conclusion:**

This integrative eQTL and Mendelian randomization analysis provides evidence of a positive causal association between 14 key co-expressed genes and mesothelioma genetically. These disease critical genes are implicated in correlations with biological processes and infiltrated immune cells related to mesothelioma. Moreover, our study lays a theoretical foundation for further research into the mechanisms of mesothelioma and potential clinical applications.

**Supplementary Information:**

The online version contains supplementary material available at 10.1186/s12931-025-03219-4.

## Introduction

Mesothelioma is an uncommon and treatment-resistant form of cancer that develops from serosal mesothelial cells. Its onset is frequently linked to exposure to asbestos. With the implementation of bans on asbestos, it is anticipated that the rates of both incidence and mortality related to mesothelioma will reach their highest levels in the middle of the 21st century [1]. Additionally, mesothelioma is characterized by the frequent loss or mutation of tumor suppressor genes such as cyclin dependent kinase inhibitor 2 A (CDKN2A), BRCA1 associated protein 1 (BAP1), neurofibromin 2 (NF2), and cullin 1 (CUL1) [2]. Due to the difficulty of early diagnosis and effective treatment with traditional methods, mesothelioma is often detected at an advanced stage, with a median survival time of less than one year and a poor prognosis [3]. Hence, the identification of a set of reliable biomarkers for mesothelioma holds considerable importance in facilitating early detection and the implementation of clinical immunotherapy [4].

Expression quantitative trait loci (eQTL) are genomic regions statistically associated with variations in gene expression levels. By identifying how specific genetic variants influence gene activity, eQTLs link genetic variation to complex traits and diseases [5]. Mendelian randomization (MR) is a method that uses genetic variation to determine the causal relationship between observed risk factors and disease outcomes. By leveraging genetic variation as a “natural experiment,” MR offers a more efficient way to test causality, effectively minimizing the influence of confounding factors [6, 7].

**Fig. 1:**
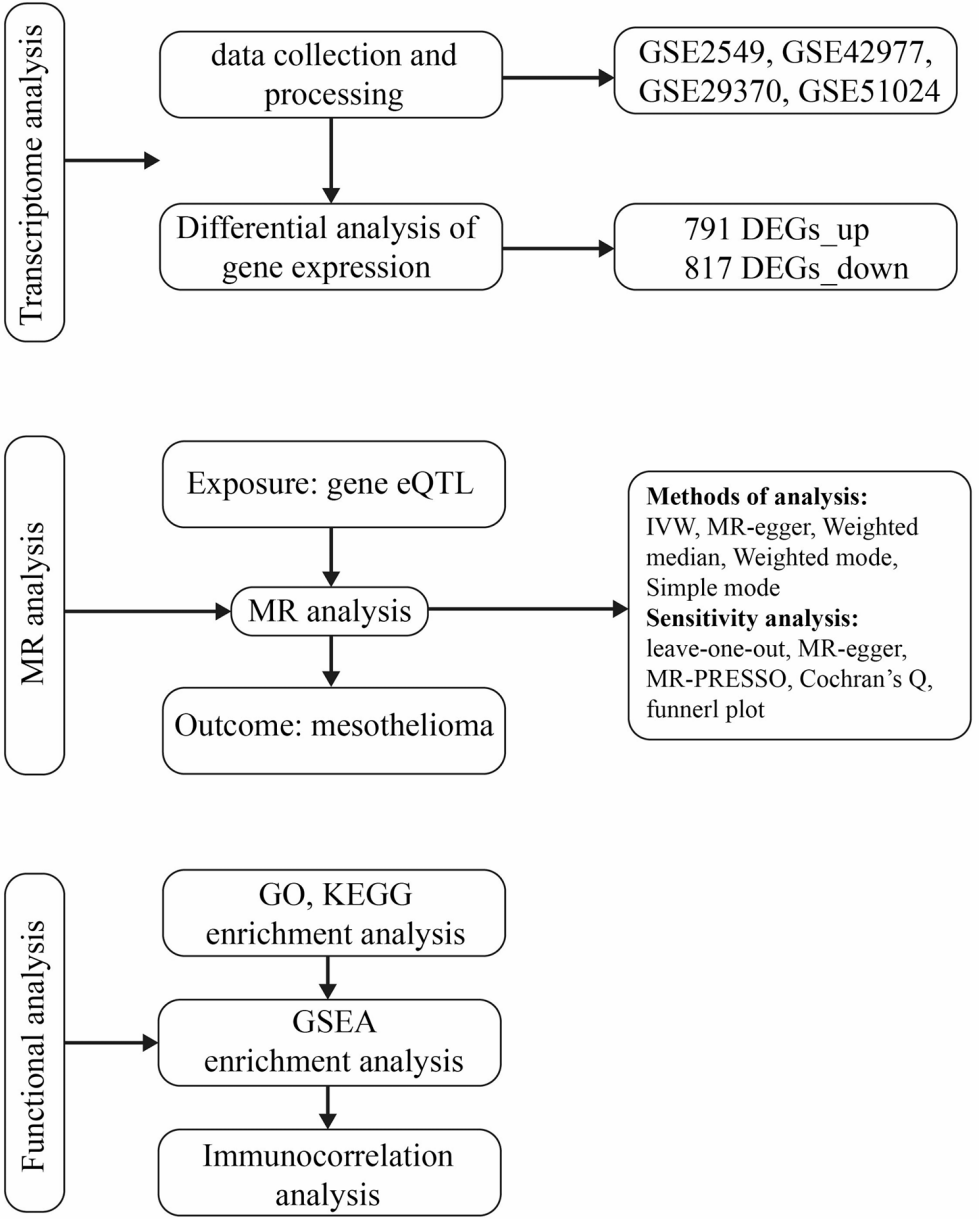
Workflow diagram of this study

In this research, MR was employed to investigate the correlation between human whole-genome eQTL data and mesothelioma Genome-Wide Association Study (GWAS) data [8]. Additionally, mesothelioma transcriptome data sourced from the Gene Expression Omnibus (GEO) database was integrated to pinpoint significant genes. Analyses conducted using Gene Ontology (GO), Kyoto Encyclopedia of Genes and Genomes (KEGG), and Gene Set Enrichment Analysis (GSEA) methods provided insights into the pathophysiological implications of key genes in the development and progression of mesothelioma. Using the cell immune infiltration analysis algorithm “CIBERSORT,” we constructed an immune infiltration model for mesothelioma and analyzed the correlation between the expression levels of key genes and immune infiltrating cells in mesothelioma. Ultimately, we validated the differential expression of key genes using external data from the Cancer Genome Atlas (TCGA) and GEO database, enhancing the reliability of our findings. This research seeks to investigate potential molecular indicators of mesothelioma and assess their biological implications on the disease. The findings are intended to guide forthcoming studies on the mechanisms of mesothelioma and the development of immunotherapeutic approaches (Fig. 1).

## Materials and methods

### Data collection and processing

Four human mesothelioma transcriptome datasets were obtained from the GEO database (https://www.ncbi.nlm.nih.gov/geo/), including GSE2549 (45 mesothelioma samples and 9 normal samples) [9], GSE42977 (39 mesothelioma samples and 9 normal samples) [10], GSE29370 (21 mesothelioma samples and 1 normal sample), and GSE51024 (55 mesothelioma samples and 41 normal samples) [11]. The GSE2549, GSE42977, and GSE29370 datasets were integrated into a training set using the “limma” and “sva” packages in R software (version 4.3.1). Batch correction was conducted through principal component analysis (PCA). The training dataset comprised 105 samples of mesothelioma and 19 samples of normal mesothelial tissue. The GSE51024 dataset was utilized as a validation set for further confirmation (Table 1). Furthermore, transcriptome expression data from 86 mesothelioma samples (TCGA-MESO.htseq_fpkm.tsv) and 110 normal lung tissue samples (TCGA-LUAD.htseq_fpkm.tsv and TCGA-LUSC.htseq_fpkm.tsv) were obtained from TCGA database (https://portal.gdc.cancer.gov/) for external validation.

**Table 1:** Microarray data details in this study

GEO accession	Platform	Mesothelioma	Normal	Data type
GSE2549	GPL96-57554	45	9	Training
GSE42977	GPL570-55999	39	9	Training
GSE29370	GPL6790-11603	21	1	Training
GSE51024	GPL570-55999	55	41	Testing

## Differential gene expression analyses (DEGs)

In the R programming environment, the “limma” and “dplyr” packages were utilized to detect differentially expressed genes (DEGs) in mesothelioma samples compared to normal controls, with statistical significance determined at a threshold of *P* < 0.05 and|Fold Change| < 0.585. Following this analysis, visual representations of the DEGs were generated using the “ggplot2” package and the “pheatmap” package to draw volcano plot and heatmap.

## Mendelian randomization

### Mendelian randomization data sources

In the R programming software, we employed Mendelian randomization utilizing GWAS data to investigate the causal relationship between genes in the human genome and mesothelioma. We downloaded the eQTL dataset from the OpenGWAS database (https://gwas.mrcieu.ac.uk/) to serve as the exposure GWAS data, encompassing 19,942 genes. We acquired the finn-b-C3_MESO_THELIOMA dataset from the FINNGEN database as the outcome GWAS data for mesothelioma, comprising 218,792 samples and 16,380,466 single nucleotide polymorphisms (SNPs). Both the exposure and outcome GWAS datasets for mesothelioma are sourced from European populations, ensuring uniformity and coherence of the data. The MR analysis conducted in this research utilized publicly GWAS summary data, thus no additional ethical approval or informed consent was required.

## Selection of instrumental variables (IVs)

The selection of instrumental variables (IVs) was conducted by applying specific criteria. This involved utilizing the “TwoSampleMR” package to assess the eQTL dataset of each gene and identifying SNPs that exhibited a strong association with gene expression levels (*P* < 5e-08) [12]. Set the linkage disequilibrium condition to r² < 0.001, and a physical distance threshold of 10,000 base pairs. To determine the presence of weak variable bias, it is recommended to compute the F-statistic of exposure, with instrumental variables having an F-value below 10 indicating the existence of such bias, which should be excluded [13].

### Mendelian randomization analysis

Following the identification of IVs with effective causal detection capability, a two-sample MR analysis was conducted on the exposure and outcome using the “TwoSampleMR” and “VariantAnnotation” packages [14, 15]. Five analytical methods were employed for the MR analysis: Inverse-variance weighted (IVW), MR-Egger, weighted median, weighted mode, and simple mode, with IVW being the primary method [16]. An assumption in MR analysis is that the selected IVs can only affect the outcome through exposure. Therefore, to make the results between exposure and outcome more stable and reliable, testing for horizontal pleiotropy is necessary. In this research investigation, the MR-Egger intercept test was employed to evaluate horizontal pleiotropy, with a significance threshold set at *P* < 0.05 [17]. Deviation of the MR-Egger intercept from zero indicates the existence of horizontal pleiotropy, which raises concerns about the reliability of the results. Such deviation suggests that the SNPs under consideration are linked solely to the exposure variable and are not influenced by other potential confounding factors. Additionally, due to variations in the selected population and experimental conditions, there may be heterogeneity among samples, potentially biasing the results. Therefore, Cochran’s Q test was used to detect heterogeneity (*P* > 0.05), and a leave-one-out analysis was performed to assess the consistency of the pleiotropy test results.

## Screening and validation of key genes for mesothelioma

Based on the analysis results in MR, genes exhibiting heterogeneity and pleiotropy were filtered out. The genes associated with a significance level of *P* < 0.05 were divided into high-risk genes (OR > 1.0) and low-risk genes (OR < 1.0) based on the odds ratio (OR) value. The “VennDiagram” package was utilized to intersect the key associated genes with the upregulated and downregulated genes of DEGs, and a Venn diagram was drawn. Using the “grid,” “readr,” and “forestploter” packages, we screened and plotted a forest plot of strongly associated genes based on the results of IVW and weighted median analyses. The “circlize” package was used to create a circular genomic map to display the chromosomal locations of key genes [18]. In the GSE51024 and TCGA datasets, differential expression validation of key genes was performed using the “limma,” “reshape2,” and “ggpubr” packages.

## Functional enrichment analysis of key genes

Using the “clusterProfiler,” “org.Hs.eg.db,” and “enrichplot” packages, we conducted GO functional and KEGG pathway enrichment analyses on key genes (*P* < 0.05, adjuested *P* < 0.05). This analysis encompassed the examination of cellular components (CC), molecular functions (MF), biological processes (BP), and signaling pathways associated with the identified key genes, followed by the creation of graphical representations in the form of bubble charts. The GSEA analysis for each key gene’s high and low expression groups was conducted on the GEO training set expression matrix data by utilizing the “c2.cp.kegg.Hs.symbols” dataset sourced from the MSigDB database.

### Immune cell infiltration and immune correlation analysis

Immune cell infiltration levels in the control and experimental groups of the GEO mesothelioma dataset were examined using the “LM22” file and the “CIBERSORT” package. Statistical significance was determined at a threshold of *P* < 0.05 with 1000 permutations. Visualization of the results was achieved through the generation of boxplots and bar plots utilizing the “corrplot” and “ggplot2” packages. Furthermore, the relationship between mesothelioma immune infiltrating cells and key genes was investigated using the “linkET” package.

### Statistical analysis

Quantitative data is represented using the mean ± standard deviation (SD). Kaplan-Meier method was used to plotted survival curve and log-rank test was performed. In consideration of other confounding factors and the impact of a suppressor effect, variables with a significant value of *P* < 0.1 were subjected to a multivariate Cox proportional hazard analysis and further screened by forward selection method to evaluate their independent effect. The Cox proportional hazard regression models were used for multivariate analysis, and the relative risks of dying were expressed as adjusted OR and corresponding 95% confidence intervals (CIs). Pearson’s analysis was used to analyze the correlation between disease critical genes and infiltrated immune cells. Statistical significance was determined by the independent paired *t*-test or one-way analysis of variance with Dunnett’s post hoc analysis using SPSS software (version 24.0; IBM Corp.). *P < 0.05, **P < 0.01, ***P < 0.001.

## Results

### DEGs identification

**Fig. 2:**
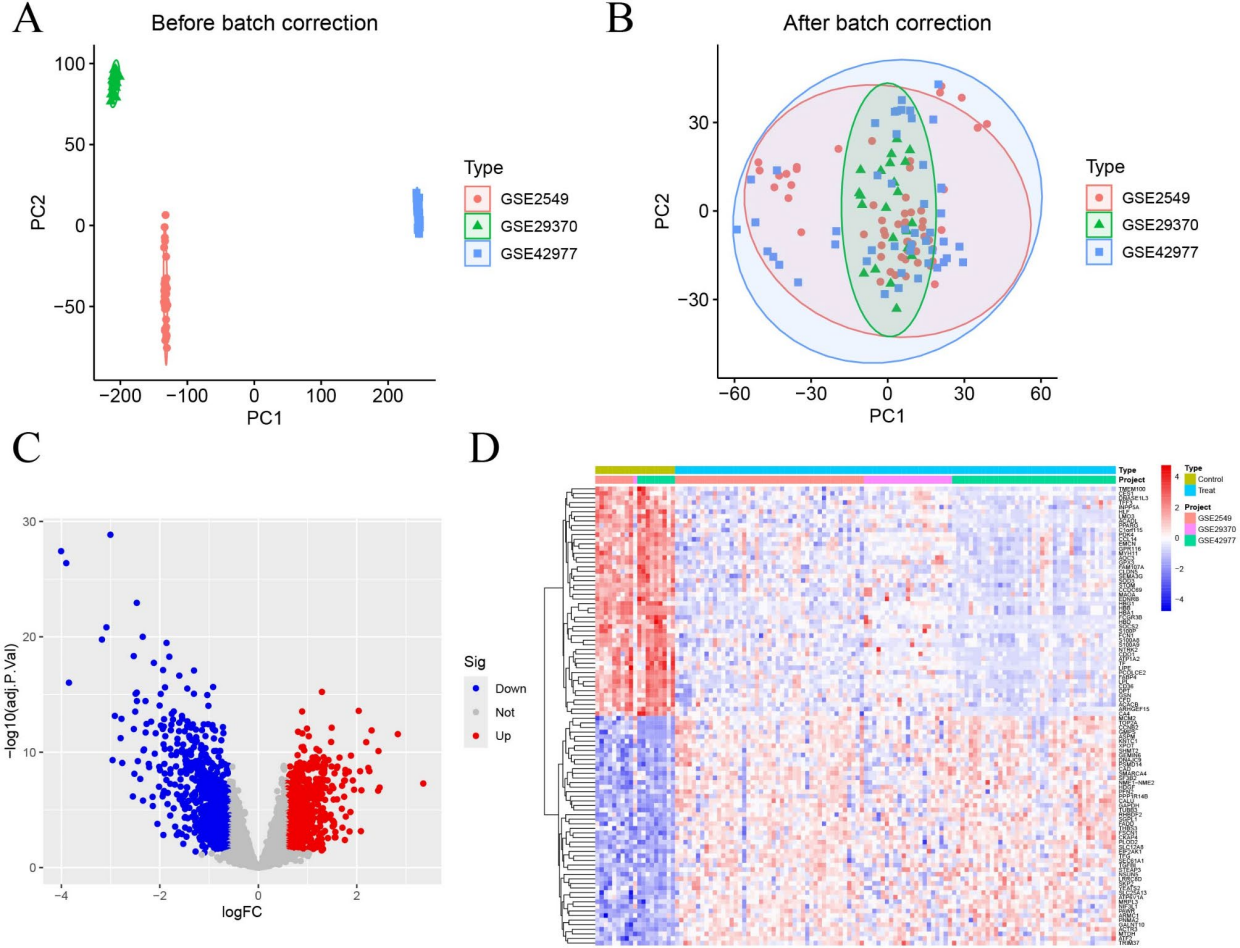
Batch correction and variance analysis. **(A)** Before the batch correction. **(B)** After the batch correction. **(C)** Volcano plot of differential expression genes. **(D)** Heatmap of differential expression genes

Batch correction was performed on the mesothelioma training set (Fig. 2A, B), with the training set details provided in Table 1. Differential gene expression analysis of the mesothelioma training set identified a total of 1608 DEGs, including 791 upregulated and 817 downregulated genes (Supplementary Table S1), as displayed in the volcano plot and heatmap (Fig. 2C, D).

### Instrumental variables selection (IVs) and MR analysis

**Fig. 3:**
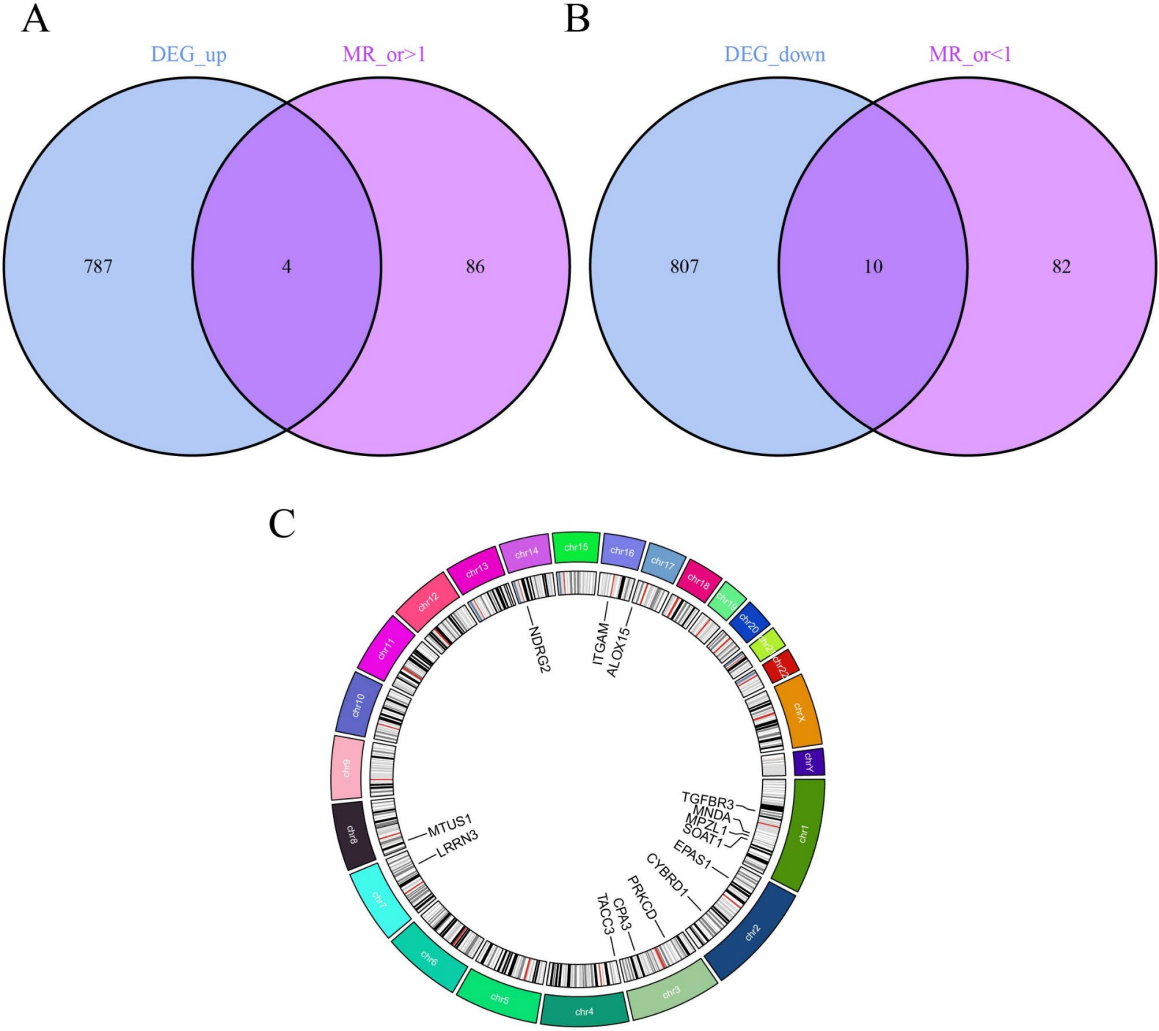
Screening and localization of critical genes. **(A)** Disease upregulated DEGs are intersected with genes with OR values greater than one in the MR results. **(B)** Disease downregulated DEGs are intersected with genes with OR values less than one in the MR results. **(C)** Position of disease-critical genes on human chromosomes

**Fig. 4:**
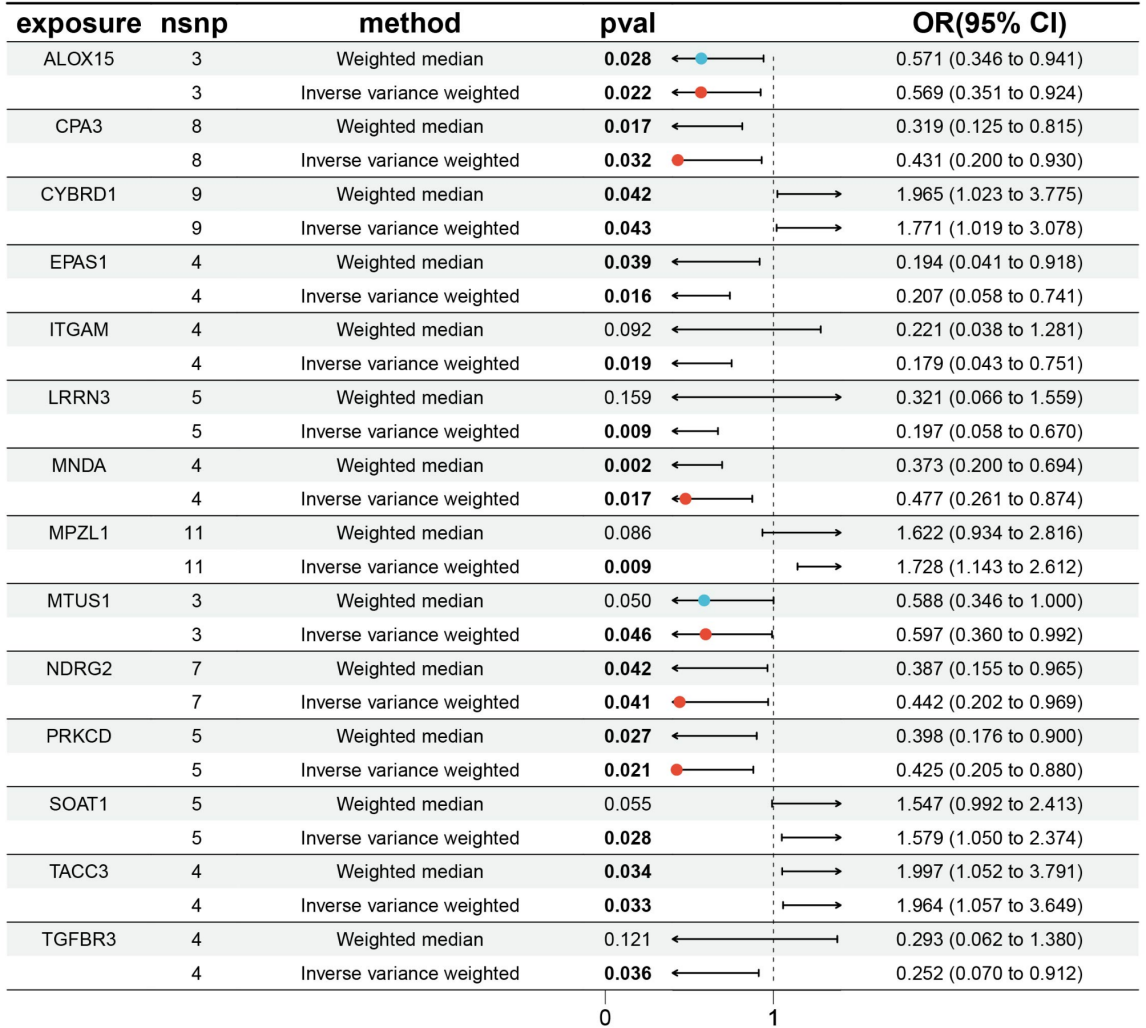
Disease critical genes causally associated with mesothelioma

Following the elimination of linkage disequilibrium and weak IVs, a total of 26,152 SNPs were pinpointed as robust IVs strongly linked to genes (Supplementary Table S2). Using two-sample MR analysis to evaluate the effect of each SNP on mesothelioma, sensitivity analyses were conducted on SNPs with IVW *P* < 0.05 and without pleiotropy. Consequently, 182 genes associated with mesothelioma were identified (Supplementary Table S3), including 90 high-risk genes and 92 low-risk genes. By intersecting high-risk and low-risk genes identified from MR analysis with upregulated and downregulated DEGs, respectively, we identified 14 key co-expressed genes. The human chromosomal locations of these 14 genes are shown in Fig. 3. Among them, the genes myelin protein zero like 1(MPZL1), sterol O-acyltransferase 1 (SOAT1), transforming acidic coiled-coil containing protein 3 (TACC3), cytochrome b reductase 1 (CYBRD1) are associated with high risk of mesothelioma, while the genes transforming growth factor beta receptor 3 (TGFBR3), NDRG family member 2 (NDRG2), endothelial PAS domain protein 1 (EPAS1), carboxypeptidase A3 (CPA3), myeloid cell nuclear differentiation antigen (MNDA), protein kinase C delta (PRKCD), microtubule associated scaffold protein 1 (MTUS1), arachidonate 15-lipoxygenase (ALOX15), leucine rich repeat neuronal 3 (LRRN3), integrin subunit alpha M (ITGAM) are low-risk genes for mesothelioma (Fig. 4). The MR analysis of the causal relationship between the 14 key genes and mesothelioma revealed that the expression levels of MPZL1 (OR = 1.728; 95% CI: [1.143–2.612]; *P* = 0.009), SOAT1 (OR = 1.579; 95% CI: [1.050–2.374]; *P* = 0.028), TACC3 (OR = 1.964; 95% CI: [1.057–3.649]; *P* = 0.033), and CYBRD1 (OR = 1.771; 95% CI: [1.019–3.078]; *P* = 0.043) were positively correlated with mesothelioma. Conversely, the expression levels of TGFBR3 (OR = 0.252; 95% CI: [0.070–0.912]; *P* = 0.036), NDRG2 (OR = 0.442; 95% CI: [0.202–0.969]; *P* = 0.041), EPAS1 (OR = 0.207; 95% CI: [0.058–0.741]; *P* = 0.016), CPA3 (OR = 0.431; 95% CI: [0.200–0.930]; *P* = 0.032), MNDA (OR = 0.477; 95% CI: [0.261–0.874]; *P* = 0.017), PRKCD (OR = 0.425; 95% CI: [0.205–0.880]; *P* = 0.021), MTUS1 (OR = 0.597; 95% CI: [0.360–0.992]; *P* = 0.046), ALOX15 (OR = 0.569; 95% CI: [0.351–0.924]; *P* = 0.022), LRRN3 (OR = 0.197; 95% CI: [0.058–0.670]; *P* = 0.009), and ITGAM (OR = 0.179; 95% CI: [0.043–0.751]; *P* = 0.019) were negatively correlated with mesothelioma.

### Gene sensitivity analysis and differential expression verification

**Fig. 5:**
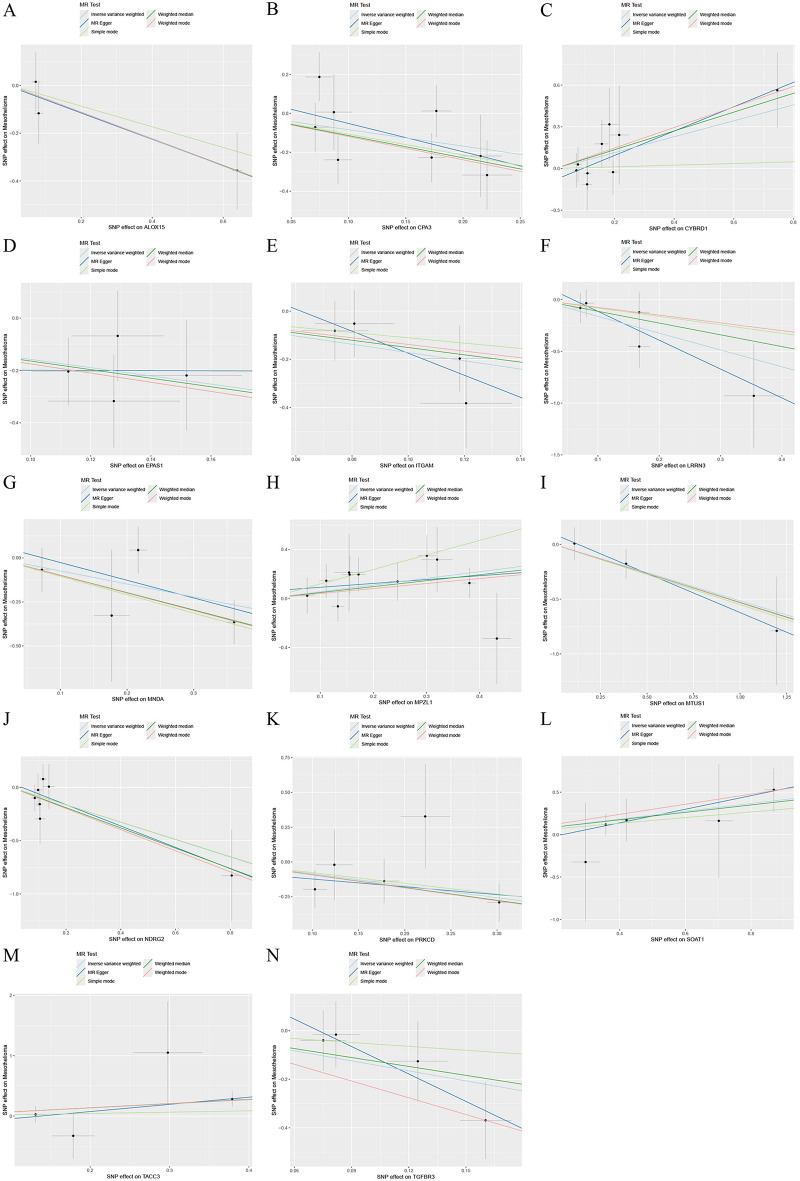
Scatterplot of MR analysis of the association between mesothelioma critical genes and mesothelioma. **(A)** Scatterplot of MR analysis of ALOX15. **(B)** Scatterplot of MR analysis of CPA3. **(C)** Scatterplot of MR analysis of CYBRD1. **(D)** Scatterplot of MR analysis of EPAS1. **(E)** Scatterplot of MR analysis of ITGAM. **(F)** Scatterplot of MR analysis of LRRN3. **(G)** Scatterplot of MR analysis of MNDA. **(H)** Scatterplot of MR analysis of MPZL1. **(I)** Scatterplot of MR analysis of MTUS1. **(J)** Scatterplot of MR analysis of NDRG2. **(K)** Scatterplot of MR analysis of PRKCD. **(L)** Scatterplot of MR analysis of SOAT1. **(M)** Scatterplot of MR analysis of TACC3. **(N)** Scatterplot of MR analysis of TGFBR3

**Fig. 6:**
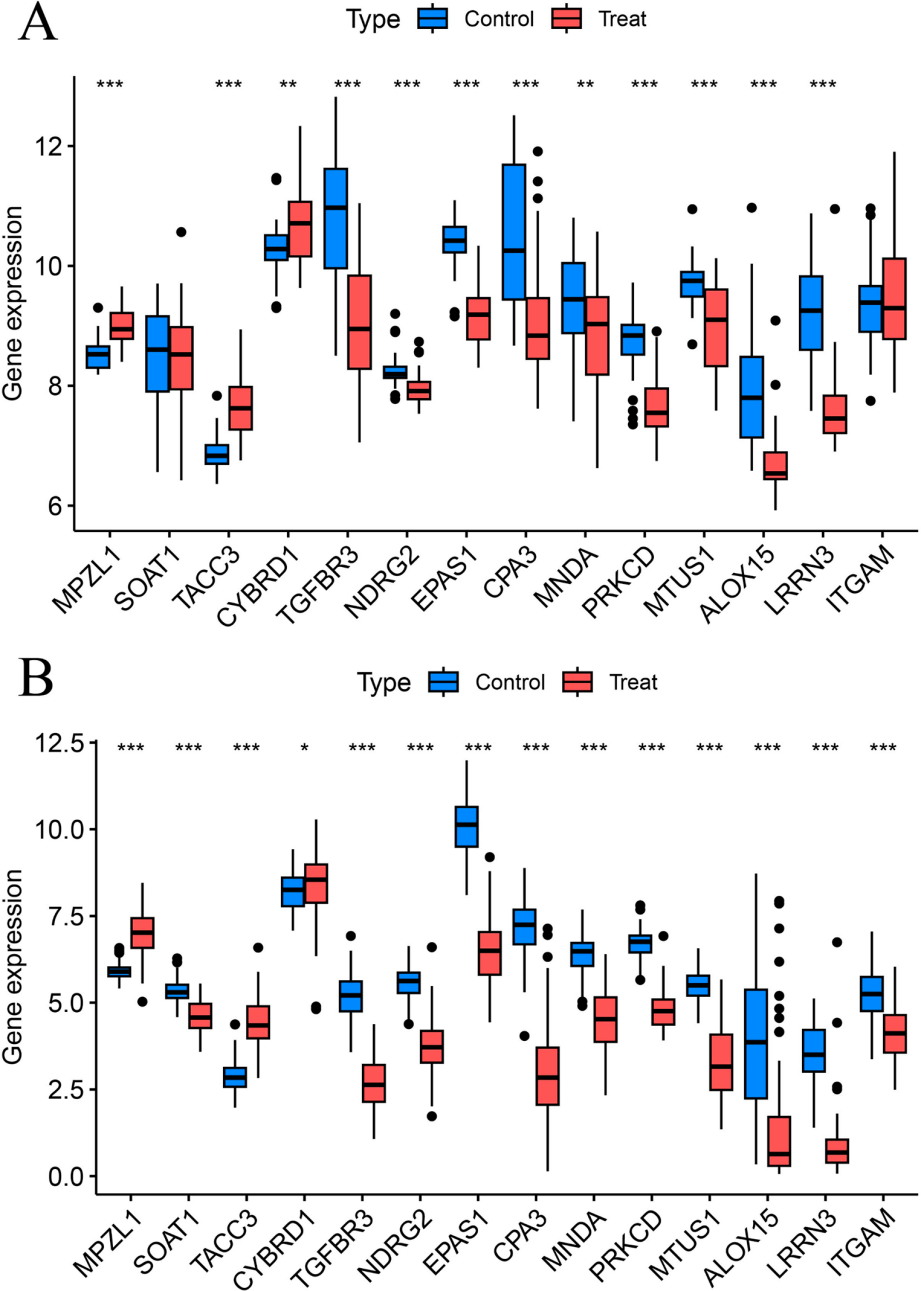
Expression validation of disease critical genes in GEO testing group and TCGA database. **(A)** GEO testing group, **(B)** TCGA database. *P < 0.05, **P < 0.01, ***P < 0.001. TCGA: The Cancer Genome Atlas. GEO: Gene Expression Omnibus.

We conducted sensitivity analyses on 14 key mesothelioma genes using MR-Egger regression and Cochran’s test. The results indicated no heterogeneity or pleiotropy, confirming the reliability of our findings (Table 2). The funnel plot analysis indicated that no individual SNP affected the outcome, implying the absence of directional pleiotropy for individual SNP non-violation and bias estimation. The leave-one-out analysis further confirmed the absence of horizontal pleiotropy, demonstrating the robustness and reliability of our analytical methods and results (Fig. 5, Supplementary Fig. 1,2). Furthermore, this research examined the variations in expression of 14 pivotal genes in mesothelioma by utilizing the validation set GSE51024 dataset and the TCGA database. The findings indicated notable distinctions in the levels of expression of these critical genes in mesothelioma, thereby validating their differential expression (Fig. 6).

**Table 2:** Sensitivity analysis of mesothelioma critical genes

Gene	*P*_MR−Egger_	*P*_MR−Egger. Q_	*P*_IVW.Q_
ALOX15	0.969	0.477	0.776
CPA3	0.536	0.261	0.311
CYBRD1	0.347	0.911	0.882
EPAS1	0.825	0.598	0.779
ITGAM	0.477	0.707	0.694
LRRN3	0.412	0.659	0.644
MNDA	0.729	0.213	0.342
MPZL1	0.588	0.668	0.724
MTUS1	0.663	0.930	0.839
NDRG2	0.703	0.686	0.776
PRKCD	0.753	0.457	0.606
SOAT1	0.549	0.937	0.929
TACC3	0.536	0.348	0.442
TGFBR3	0.357	0.852	0.631

### Functional enrichment analysis of key genes

**Fig. 7:**
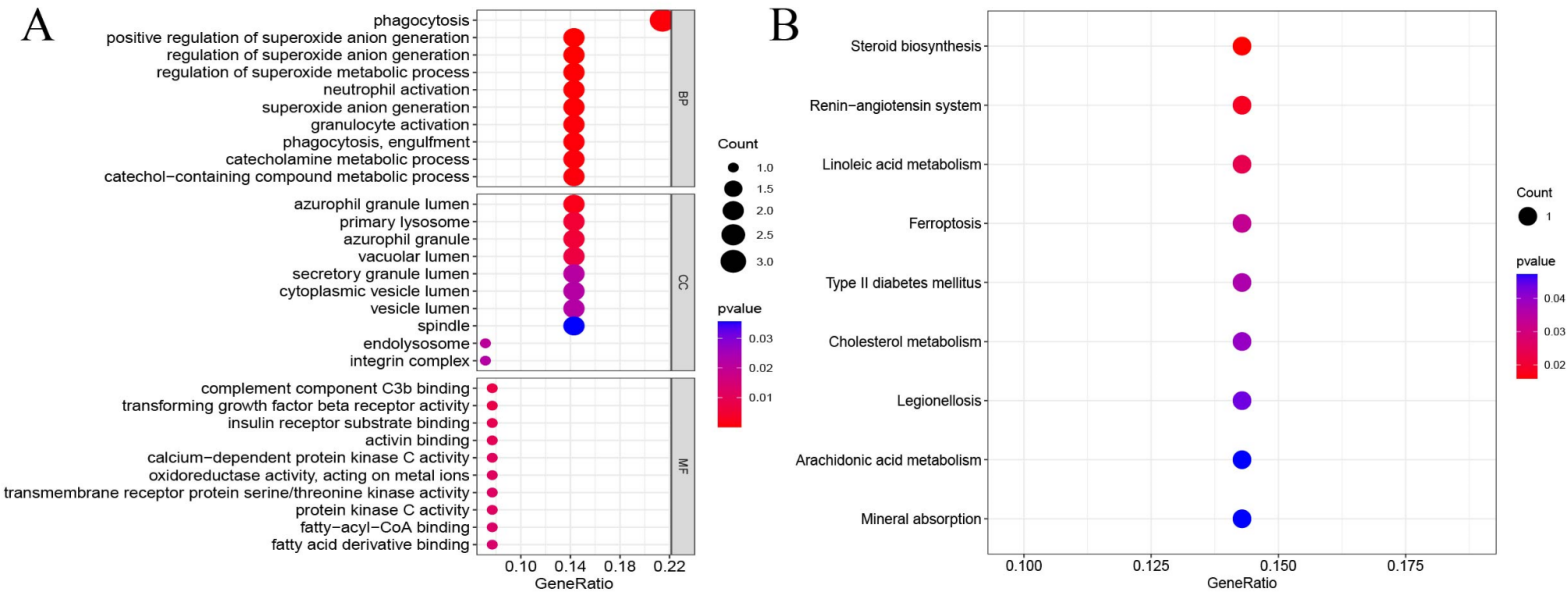
Functional enrichment analysis of critical genes. **(A)** GO enrichment analysis of mesothelioma critical genes. **(B)** KEGG enrichment analysis of mesothelioma critical genes

The analysis of GO for the identified key genes demonstrated notable enrichment in various biological processes, including phagocytosis, superoxide anion generation, superoxide metabolism, and neutrophil activation. Furthermore, these genes exhibited enrichment in cellular components such as primary lysosomes, azurophilic granules lumen, and cytoplasmic vesicles. Additionally, they were found to be enriched in molecular functions such as sulfur compound binding, amide binding, and serine/threonine kinase activity (Fig. 7A, supplementary table S4). The KEGG analysis of the key genes revealed their enrichment in pathways such as ferroptosis, steroid biosynthesis, cholesterol metabolism, and the renin-angiotensin system (Fig. 7B, supplementary table S5).

**Fig. 8:**
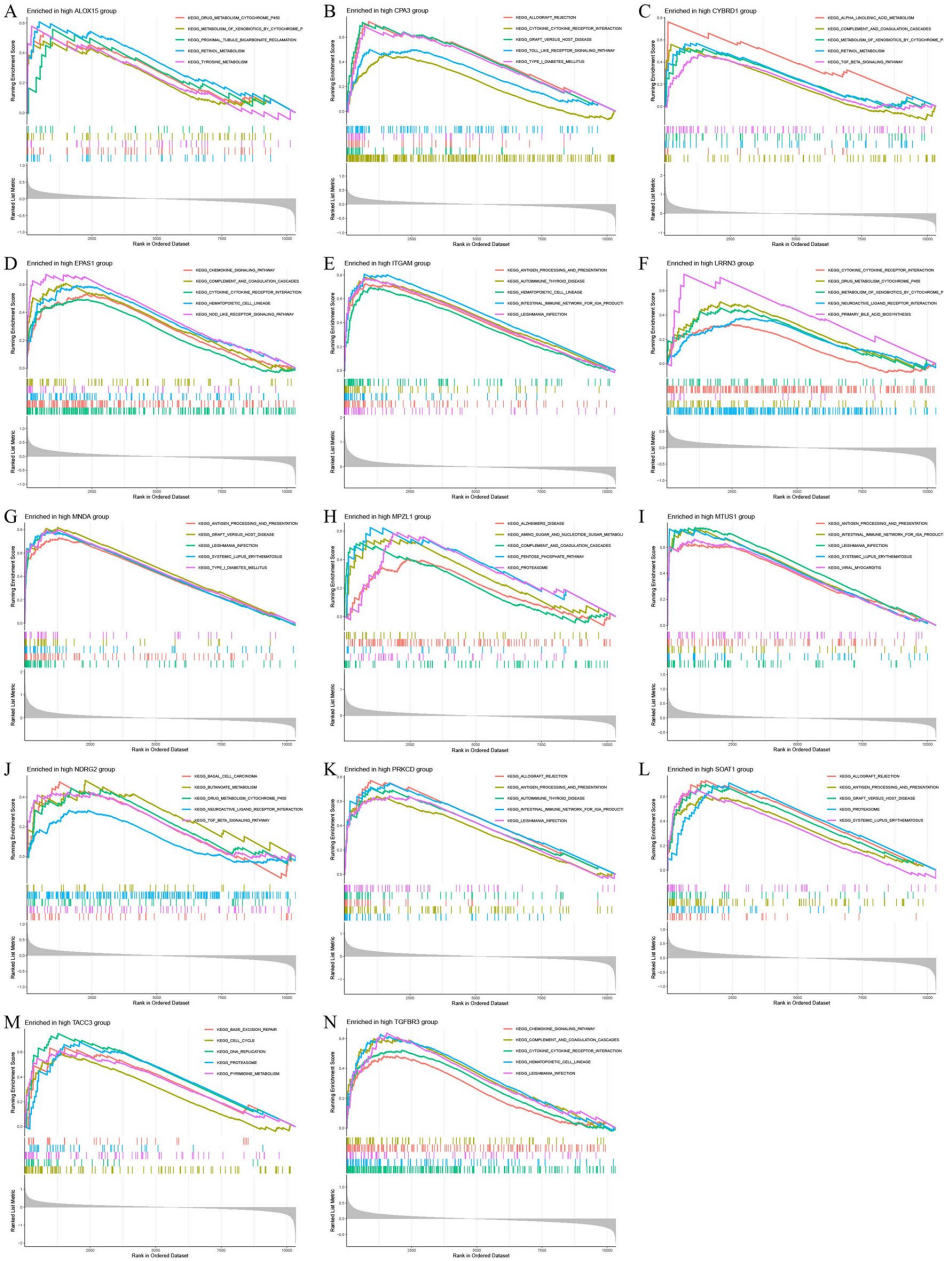
GSEA enrichment analysis of disease critical genes in mesothelioma. **(A)** GSEA enrichment results of ALOX15 high expression group. **(B)** GSEA enrichment results of CPA3 high expression group. **(C)** GSEA enrichment results of CYBRD1 high expression group. **(D)** GSEA enrichment results of EPAS1 high expression group. (E) GSEA enrichment results of ITGAM high expression group. **(F)** GSEA enrichment results of LRRN3 high expression group. **(G)** GSEA enrichment results of MNDA high expression group. (H) GSEA enrichment results of MPZL1 high expression group. **(I)** GSEA enrichment results of MTUS1 high expression group. **(J)** GSEA enrichment results of NDRG2 high expression group. **(K)** GSEA enrichment results of PRKCD high expression group. **(L)** GSEA enrichment results of SOAT1 high expression group. **(M)** GSEA enrichment results of TACC3 high expression group. **(N)** GSEA enrichment results of TGFBR3 high expression group

### The GSEA enrichment analysis of key genes

**Fig. 9:**
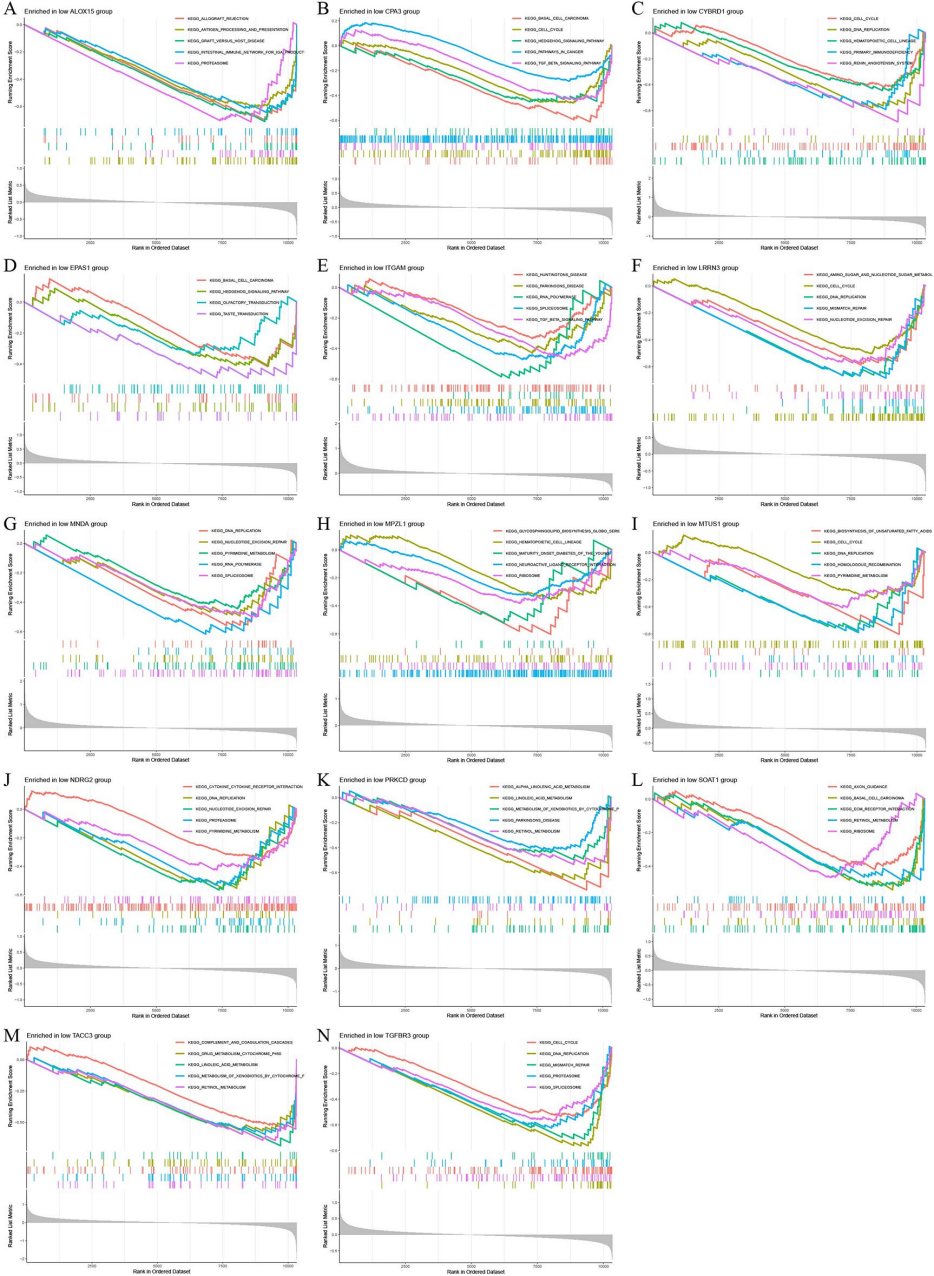
GSEA enrichment analysis of disease critical genes in mesothelioma. **(A)** GSEA enrichment results of ALOX15 low expression group. **(B)** GSEA enrichment results of CPA3 low expression group. **(C)** GSEA enrichment results of CYBRD1 low expression group. **(D)** GSEA enrichment results of EPAS1 low expression group. **(E)** GSEA enrichment results of ITGAM low expression group. **(F)** GSEA enrichment results of LRRN3 low expression group. **(G)** GSEA enrichment results of MNDA low expression group. **(H)** GSEA enrichment results of MPZL1 low expression group. **(I)** GSEA enrichment results of MTUS1 low expression group. **(J)** GSEA enrichment results of NDRG2 low expression group. (K) GSEA enrichment results of PRKCD low expression group. **(L)** GSEA enrichment results of SOAT1 low expression group. (M) GSEA enrichment results of TACC3 low expression group. (N) GSEA enrichment results of TGFBR3 low expression group

To investigate the enrichment differences of key genes in high and low expression groups in mesothelioma, gene set enrichment analysis was conducted on the key genes. The results of GSEA showed that the antigen processing and presentation signaling pathway had the highest enrichment level in the high expression group, followed by significant enrichment in the cytokine-cytokine receptor interaction and proteasome signaling pathways (Fig. 8). The key genes were most enriched in the cell cycle signaling pathway in the low expression group, followed by the proteasome, spliceosome and DNA replication signaling pathways (Fig. 9). These findings suggest that alterations in the expression levels of key genes may have an impact on the occurrence and development of mesothelioma at the levels of protein synthesis and metabolism, protein interactions, and even the cell cycle.

### Analysis of immune cell infiltration levels and their association with key genes in mesothelioma

**Fig. 10:**
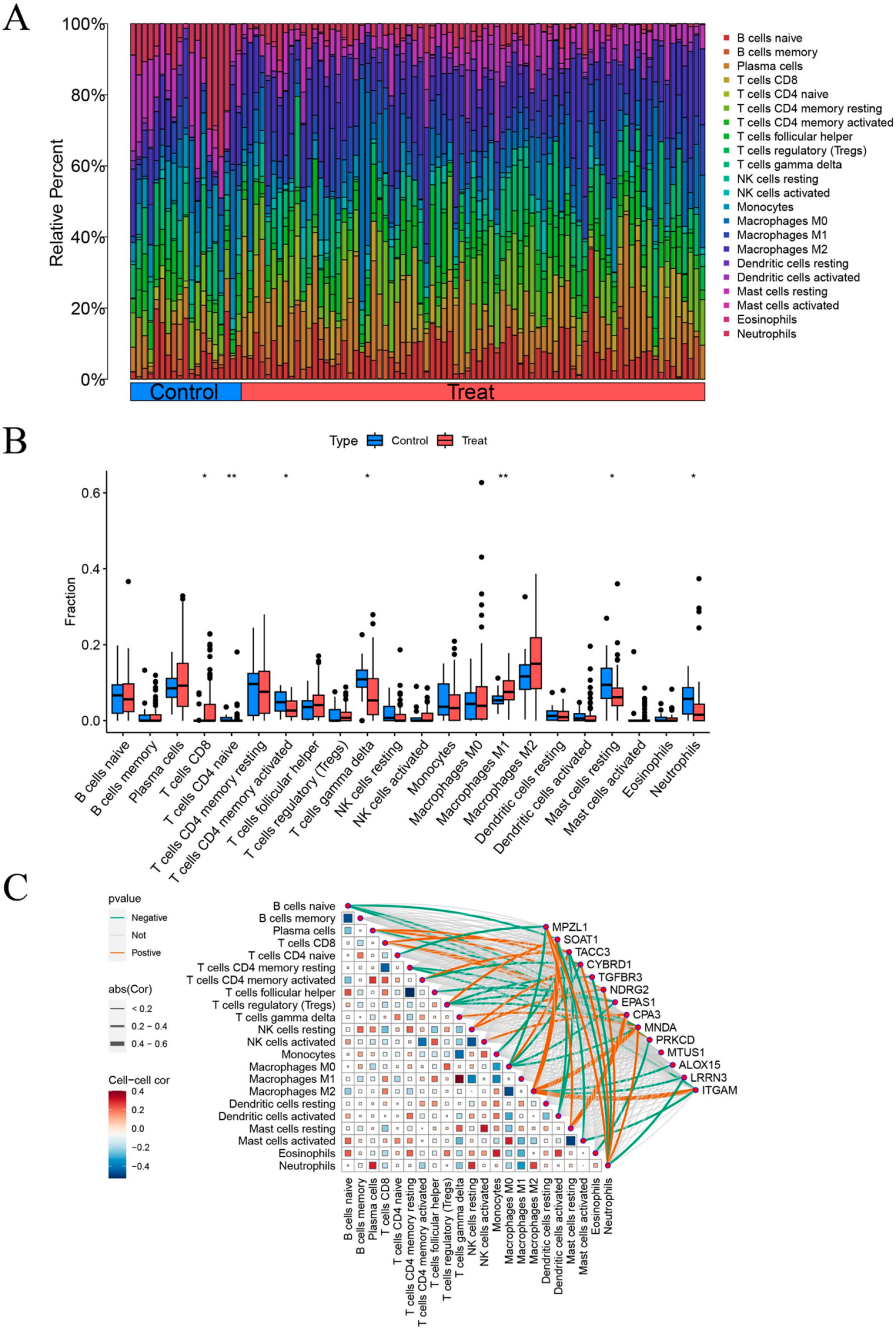
Immune infiltration analyses, correlations between disease critical genes and infiltrating immune cell types. **(A)** Stacked histogram of the proportions of immune infiltration cells between control and mesothelioma groups. **(B)** Box plot of the infiltration level of immune cells between control and mesothelioma groups. **(C)** Correlation analysis between disease critical genes and infiltrated immune cells. *P < 0.05, **P < 0.01

Using the CIBERSORT algorithm to measure differences in immune cell infiltration between mesothelioma and normal mesothelial tissues, we obtained the immune cell infiltration profile of mesothelioma. Compared to the control group, the proportions of CD4 + naive T cells and M1 macrophages were significantly increased in mesothelioma, suggesting their potential impact on the disease (Fig. 10A.B). Additionally, to investigate the immunological characteristics of the key genes, we conducted a correlation analysis between the key genes and immune infiltrating cells (Fig. 10C). The results indicate that key genes are correlated with various immune infiltrating cells. M1 macrophages show significant positive correlations with the expression levels of SOAT1, MNDA, and ITGAM, and negative correlations with ALOX15 and LRRN3. Neutrophils exhibit positive correlations with the expression levels of MPZL1, CYBRD1, NDRG2, and MNDA, and negative correlations with TACC3, TGFBR3, and LRRN3. Additionally, the infiltration levels of T cells gamma delta significantly positively correlate with M1 macrophage infiltration levels. In contrast, the infiltration level of T cell follicular helper is significantly negatively correlated with T cell CD4 memory resting. The above research discovered that certain key genes interact with immune cells in mesothelioma. Specifically, the genes MNDA, TACC3, and CYBRD1 are significantly associated with infiltration of various immune cells.

## Discussion

Mesothelioma is a rare, aggressive, and difficult-to-treat malignant tumor. Most patients are diagnosed at an advanced stage, with a median survival time of only 9-12 months and a 5-year survival rate of less than 10%. Consequently, mesothelioma ranks among the cancers with the poorest survival rates [19]. Asbestos is a significant causative factor in mesothelioma, inducing chromosomal and genetic damage in mammalian cells [20]. Mesothelioma is characterized by extensive chromosomal rearrangements, gene mutations, and deletions, primarily involving human chromosomes 9 (9p21), 21q, and 3 (3p21) [21]. Based on transcriptomic pathway analysis, mesothelioma cells exhibit alterations in cellular proliferation, apoptosis, differentiation, and migration. These alterations in pathways can disturb the homeostasis of normal mesothelial cells [22].

In this study, we employed MR to analyze eQTL data of 19,942 genes to investigate their causal relationship with mesothelioma. The MR analysis revealed 182 genes associated with the condition, which were classified into high-risk and low-risk groups according to their OR values. By intersecting high-risk genes with upregulated DEGs, we identified four key high-risk genes associated with mesothelioma: MPZL1, SOAT1, TACC3, and CYBRD1. Conversely, intersecting low-risk genes with downregulated DEGs revealed ten key low-risk genes: LRRN3, TGFBR3, NDRG2, EPAS1, CPA3, MNDA, PRKCD, MTUS1, ALOX15, and ITGAM. This study represents the first application of MR analysis using eQTL data integrated with transcriptome data to identify pivotal risk genes in mesothelioma. Additionally, we conducted functional enrichment analysis of these key genes and explored their immune correlations. This finding has the potential to offer new insights into investigating the mechanisms underlying mesothelioma and advancing the development of immunosuppressive therapy.

Although thoracoscopic biopsy can be used for early pathological diagnosis of mesothelioma, its application is limited due to significant harm to the patients [23]. To date, as an environmental tumor associated with asbestos exposure, mesothelioma currently lacks clinically validated serum biomarkers for early diagnosis [24]. In this study, we identified the MPZL1 gene as one of the core genes with a strong causal association with mesothelioma. The MPZL1 gene is located on human chromosome 1q24.2 and contains 7 exons (Fig. 3C). MPZL1 is a member of the immunoglobulin superfamily and is also known as the concanavalin receptor. As a single transmembrane protein, MPZL1’s intracellular domain contains two immunoreceptor tyrosine-based inhibitory motifs (ITIMs) that specifically bind to the SH2 domains of the tyrosine phosphatase SHP-2/PTPN11 [25, 26]. In hepatocellular carcinoma, MPZL1 recruits SHP-2 to phosphorylate Src kinase at Tyr426, subsequently leading to cortactin phosphorylation, which promotes the migration of hepatocellular carcinoma cells [27]. Additionally, MPZL1 is highly expressed in various cancers, such as lung cancer, glioma, ovarian cancer, and gallbladder cancer [28–32]. MPZL1 promotes metastasis of non-small cell lung cancer (NSCLC) by upregulating COL11A1 and is associated with a poor prognosis in NSCLC [29]. MPZL1 can bind with β1 transforming growth factor to promote the development of lung adenocarcinoma. The upregulation of MPZL1 in lung adenocarcinoma negatively regulates the infiltration of CD8 + T cells, which is associated with resistance to immunotherapy [32]. However, in another study, MPZL1 was found to inhibit the proliferation, metastasis, invasion, and cancer stem cell characteristics of lung adenocarcinoma by activating the β-catenin/TCF-4 signaling pathway [33], highlighting the complex role of MPZL1 in tumor development.

Additionally, we discovered that the genes SOAT1, TACC3, and NDRG2 are linked to the prognosis of mesothelioma patients. Elevated levels of SOAT1 and TACC3 were connected to shorter survival times (*p* < 0.05), while increased expression of NDRG2 was related to a more favorable prognosis (Supplementary Fig. 3). The SOAT1 gene is located on human chromosome 1q25.2 (Fig. 3C) and encodes a protein that resides in the endoplasmic reticulum. As a member of the acyltransferase family, it is primarily expressed in adrenal tissues [34]. The SOAT1 protein catalyzes the synthesis of fatty acid-cholesterol esters and promotes epithelial-mesenchymal transition (EMT) in hepatocellular carcinoma by regulating cholesterol metabolism [35]. Furthermore, the SOAT1 protein can activate the PI3K/AKT signaling pathway to promote lung cancer invasiveness by downregulating intracellular free cholesterol levels [36]. According to Huang’s pan-cancer analysis, the SOAT1 gene exhibits increased expression in various cancers, such as lung adenocarcinoma, lung squamous cell carcinoma, breast cancer, hepatocellular carcinoma, and gastric cancer. It is associated with infiltration of different immune cells, including T cells, neutrophils, and macrophages, as well as co-expression with numerous immune-related genes. These findings align with our analysis of SOAT1 gene immunoreactivity [37]. TACC3 is a transforming acidic coiled-coil protein expressed in the spindle, centrosome, and nucleus, it is involved in maintaining the stability of cell mitosis [38, 39]. In breast cancer, the loss or mutation of the p53 gene can upregulate TACC3 expression via FOXM1, leading to increased centrosome amplification (CA) and enhanced invasiveness [40]. Similarly, p53 is a commonly mutated tumor suppressor gene in mesothelioma, and TACC3 could be a downstream gene involved in its pathogenesis [41]. Additionally, TACC3 can promote the EMT of gastric cancer cells through the ERK/Akt/cyclin D1 signaling pathway, which is a critical process in the metastasis of mesothelioma [42, 43]. Similarly, literature reports that high TACC3 expression is associated with tumor immune cell infiltration and poor prognosis in lung adenocarcinoma patients, serving as an independent prognostic indicator [44, 45]. The NDRG2 gene, also known as SYLD, belongs to the hydrolase superfamily and is a cytoplasmic protein that promotes neurite growth. It is downregulated in various cancers, including breast and colorectal cancer, and is considered a negative indicator of tumor metastasis [46, 47]. The expression of the NDRG2 gene is downregulated in human lung cancer tissues and is considered a favorable prognostic indicator for lung cancer [48]. Salvianolic acid B (Sal B) can induce oxidative stress and inhibit the growth and metastasis of lung cancer A549 cells through the NDRG2/PTEN signaling pathway [49]. In a separate study, overexpression NDRG2 protein via a survivin promoter suppressed the viability and invasiveness of lung cancer cells [50]. Additionally, NDRG2 can negatively regulate the progression of small cell lung cancer through the PTEN-AKT-mTOR signaling pathway [51]. Currently, there are no research reports on the roles of SOAT1, TACC3, and NDRG2 in mesothelioma. Further investigation is needed to understand their functions in the progression of mesothelioma.

However, our study has some unavoidable limitations. Firstly, mesothelioma is a rare malignant tumor, leading to a limited sample size across multiple databases, such as GEO and TCGA database. Moreover, due to the absence of normal mesothelial samples in TCGA database, we utilized gene expression matrices from normal lung tissue for comparison and validation. Secondly, the GWAS data used for MR analysis were all derived from European populations, which may limit the generalizability of the findings to other populations. Lastly, our study developed a predictive model consisting of 14 genes. To improve prediction accuracy, further analysis and screening are required to identify the most pertinent key genes, which should then be validated through experimental studies.

## Electronic supplementary material

Below is the link to the electronic supplementary material.

Supplementary Material 1

Supplementary Material 2

## Data Availability

All original data for this study are in the article or supplementary material, and all GWAS data as well as transcriptomic data are publicly available.
