# Supplementary material for: Integrative eQTL and Mendelian randomization analysis reveals key genetic markers in mesothelioma
Source: Respir Res. 2025 Apr 13;26:140. doi: 10.1186/s12931-025-03219-4 (PMC11995628; doi:10.1186/s12931-025-03219-4)
Supplement: Supplementary file 1 — Supplementary Material 1 [file 12931_2025_3219_MOESM1_ESM.docx]

Supplementary Figures


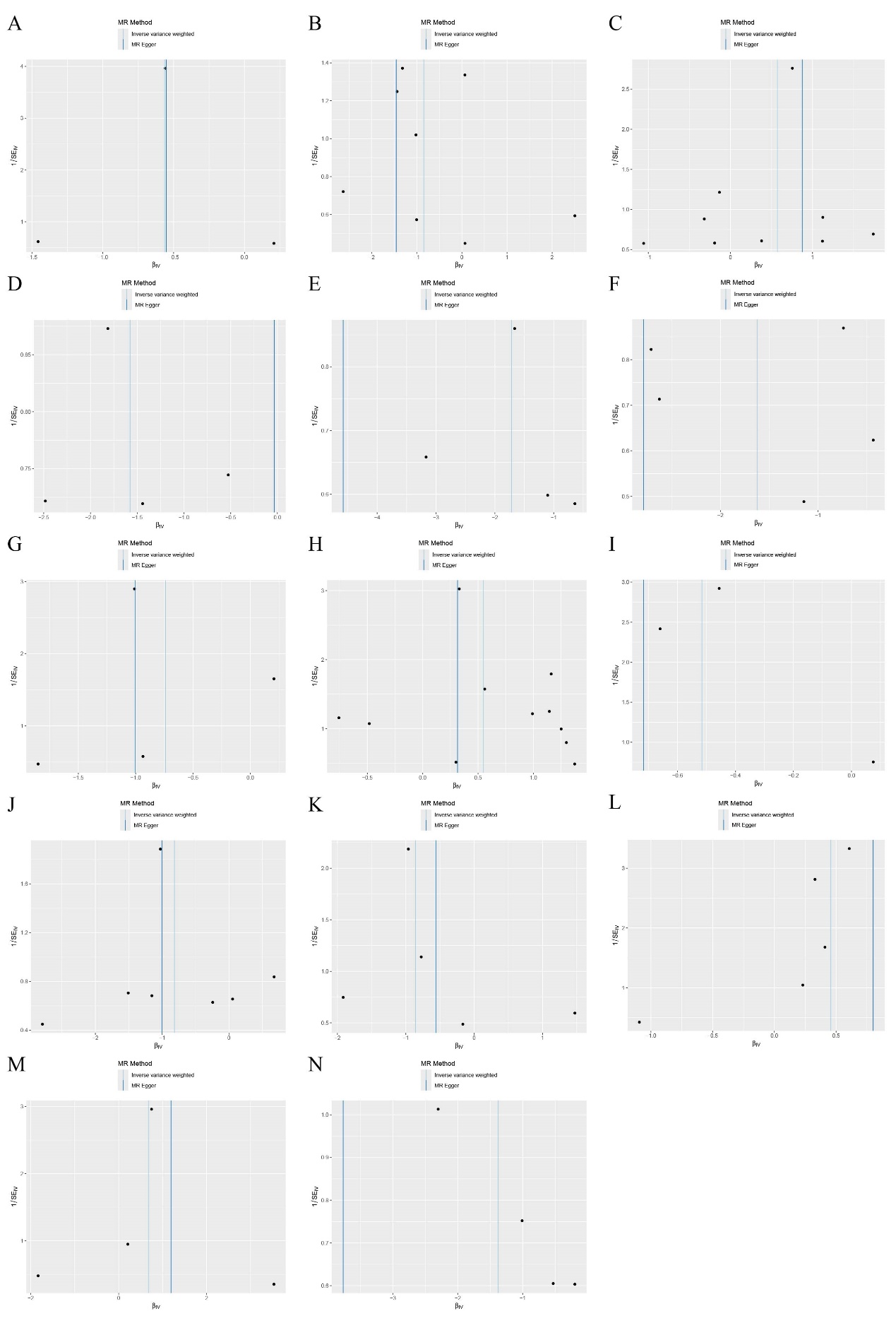


Supplementary Fig. 1 Sensitivity analysis of disease critical genes on mesothelioma. (A) Funnel plot of MR analysis of ALOX15. (B) Funnel plot of MR analysis of CPA3. (C) Funnel plot of MR analysis of CYBRD1. (D) Funnel plot of MR analysis of EPAS1. (E) Funnel plot of MR analysis of ITGAM. (F) Funnel plot of MR analysis of LRRN3. (G) Funnel plot of MR analysis of MNDA. (H) Funnel plot of MR analysis of MPZL1. (I) Funnel plot of MR analysis of MTUS1. (J) Funnel plot of MR analysis of NDRG2. (K) Funnel plot of MR analysis of PRKCD. (L) Funnel plot of MR analysis of SOAT1. (M) Funnel plot of MR analysis of TACC3. (N) Funnel plot of MR analysis of TGFBR3.


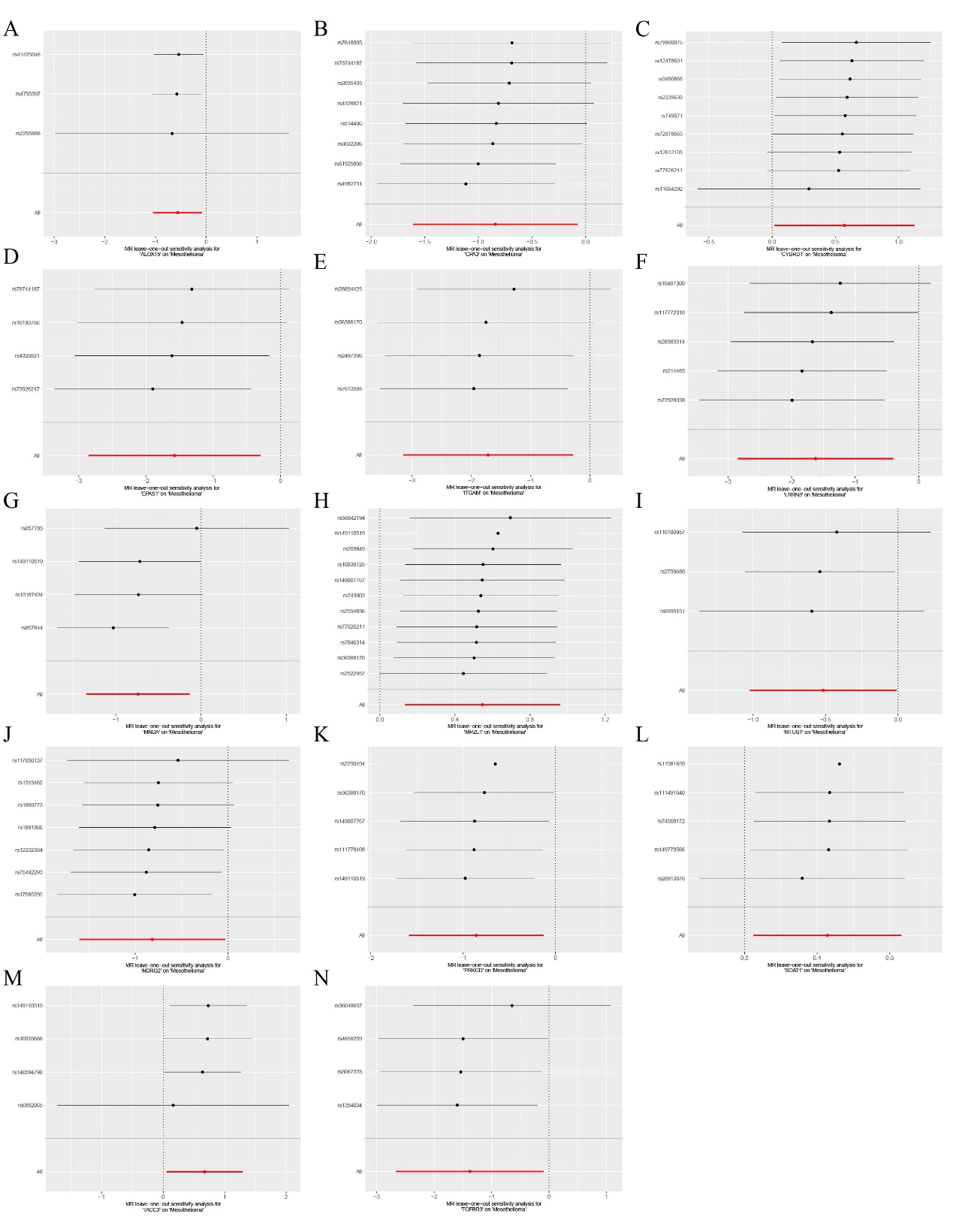
 **Supplementary Fig. 2**

Result of “leave-one-out” sensitivity analysis of the causal effect of disease critical genes on mesothelioma. **(A)** MR leave−one−out sensitivity analysis of ALOX15 in mesothelioma. **(B)** MR leave−one−out sensitivity analysis of CPA3 in mesothelioma. **(C)** MR leave−one−out sensitivity analysis of CYBRD1 in mesothelioma. **(D)** MR leave−one−out sensitivity analysis of EPAS1 in mesothelioma. **(E)** MR leave−one−out sensitivity analysis of ITGAM in mesothelioma. **(F)** MR leave−one−out sensitivity analysis of LRRN3 in mesothelioma. **(G)** MR leave−one−out sensitivity analysis for MNDA in mesothelioma. **(H)** MR leave−one−out sensitivity analysis of MPZL1 in mesothelioma. **(I)** MR leave−one−out sensitivity analysis of MTUS1 in mesothelioma. **(J)** MR leave−one−out sensitivity analysis of NDRG2 in mesothelioma. **(K)** MR leave−one−out sensitivity analysis of PRKCD in mesothelioma. **(L)** MR leave−one−out sensitivity analysis of SOAT1 in mesothelioma. **(M)** MR leave−one−out sensitivity analysis of TACC3 in mesothelioma. **(N)** MR leave−one−out sensitivity analysis of TGFBR3 in mesothelioma.


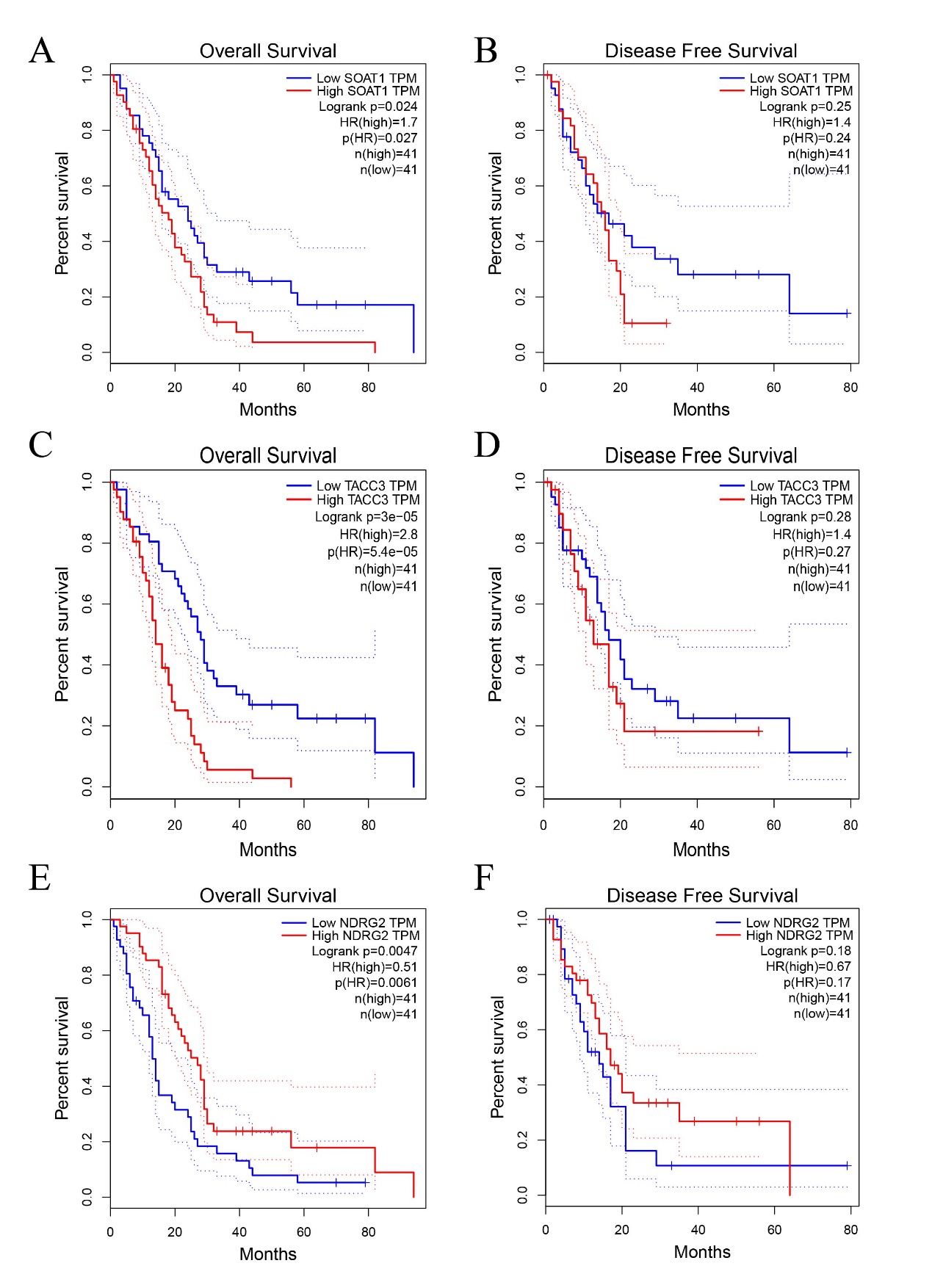


**Supplementary Fig. 3**

Results of the effects of SOAT1, TACC3, and NDRG2 genes on the prognosis of mesothelioma. **(A)** Overall survival curve of SOAT1 gene in mesothelioma. **(B)** Disease-free survival curve of SOAT1 gene in mesothelioma. **(C)** Overall survival curve of TACC3 gene in mesothelioma., **(D)** Disease-free survival curve of TACC3 gene in mesothelioma. **(E)** Overall survival curve of NDRG2 gene in mesothelioma. **(F)** Disease-free survival curve of NDRG2 gene in mesothelioma.
